# Family-Based Treatment for a Preadolescent With Avoidant/Restrictive Food Intake Disorder With Sensory Sensitivity: A Case Report

**DOI:** 10.3389/fpsyt.2020.00350

**Published:** 2020-05-08

**Authors:** Kimberly Rosania, James Lock

**Affiliations:** Department of Psychiatry and Behavioral Sciences, Stanford University School of Medicine, Stanford, CA, United States

**Keywords:** eating disorders, feeding disorders, avoidant/restrictive food intake disorder, treatment, family based treatment

## Abstract

**Background:**

Individuals with Avoidant/Restrictive Food Intake Disorder (ARFID) experience eating problems that cause persistent failure to meet appropriate nutritional and/or energy needs. These eating problems are not driven by body image concerns but rather by persistent low appetite, sensory sensitivity, or fear of aversive consequences of eating (e.g., choking or vomiting). Although increasing numbers of youth are being referred for treatment of ARFID, no evidence-based treatments yet exist for the disorder. Given family-based treatment (FBT) has demonstrated effectiveness with other pediatric eating disorders (anorexia nervosa, bulimia nervosa), a manualized version of FBT adapted for use with ARFID patients has been developed and is currently under study.

**Case Presentation:**

The following case report demonstrates how FBT was used to treat a 9-year-old patient with ARFID characterized by sensory sensitivity. Similarities and differences with FBT for anorexia nervosa are illustrated. After 17 sessions across 6 months, the patient no longer met DSM criteria for ARFID, she demonstrated major declines in measures of clinical symptoms, and she gained 2.1 kg.

**Conclusions:**

FBT for ARFID relies upon the same key interventions as FBT for AN. However, we discuss critical differences in the application of these interventions given the unique challenges of ARFID, particularly when characterized by sensory sensitivity.

## Introduction

Avoidant/Restrictive Food Intake Disorder (ARFID) is a feeding and eating disorder introduced in the DSM-5 that describes a wide range of eating difficulties which often onset in childhood (1). Individuals with ARFID experience eating problems that cause persistent failure to meet appropriate nutritional and/or energy needs. Children may present with serious nutritional deficiencies, a reliance upon liquid supplements or enteral feeding, faltering growth or failure to gain expected weight, or psychosocial impairments. However, these eating problems are not driven by body image concerns, but rather by persistent low appetite, sensory sensitivity, and/or fear of aversive consequences of eating (e.g., choking) (2). A variety of approaches have been utilized to treat ARFID, but no evidence-based treatments yet exist. Given family-based treatment (FBT) has demonstrated effectiveness with other pediatric eating disorders, an initial description of how FBT could be applied to sensory sensitive presentations of ARFID was published (3), followed by the development of a broader manual to treat all presentations of ARFID and the first randomized treatment study for this condition (4). This case report illustrates a detailed application of this novel manualized treatment (FBT-ARFID) and highlights its similarities and differences from more well-known forms of FBT.

FBT for anorexia nervosa (AN) mobilizes families to help the child overcome AN in three phases. Phase 1 focuses on weight restoration by parents taking control of the child's eating. In the first session, the therapist emphasizes the seriousness of AN in an intense scene in order to increase parents' urgency and responsibility to take on this task. The therapist takes an agnostic approach, emphasizing that the cause of AN is unknown, in order to alleviate parental guilt and redirect parents away from focusing on potential causes and toward taking action. The therapist externalizes the eating disorder, framing it as separate from the child and not under her control. The second session involves a family meal and allows the therapist to assess and intervene with family dynamics that may affect weight restoration. Phase 2 occurs when the child is eating without much resistance, there has been steady weight gain, and parents feel empowered to manage symptoms. Phase 2 focuses on helping parents return control of eating back to their child in a way that is age-appropriate and consistent with their family. Phase 3 occurs when the child is weight restored, AN behaviors are gone, and the child is managing her own eating and exercise. This phase focuses on addressing adolescent development issues which AN disrupted.

This case illustrates differences in FBT when it is applied to ARFID. Externalization of the illness and fostering a sense of urgency to take action may be more challenging. Treatment goals may focus on eating behaviors (e.g., improving variety, flexibility, speed) rather than on weight restoration. The child may provide more helpful input during treatment and be more responsive to rewards than children with AN. Finally, Phase 2 and 3 differ; Phase 2 occurs when the child is able to try new foods consistently, and there is often no Phase 3 given children with ARFID are often pre-adolescent.

## Case Report

T was a 9-year-old female who was referred to treatment by her pediatrician due to longstanding selective eating. As an infant and young toddler, T “ate everything.” However, around age two and half, T began to lose interest in some foods she had previously enjoyed and developed aversions to certain textures and tastes. By age three, her food restriction became “categorical”. For example, she would not eat fruit, soup that was not pureed completely smooth, or vegetables that were not pureed into a food she already liked. For several years, the family and pediatrician thought this was typical “picky eating” that she would “grow out of,” and the family largely accommodated it. Instead, T's dietary restriction worsened over time. Although her pediatrician felt T's weight and growth were unaffected, her limited food variety caused marked psychosocial distress and functional impairment (see **Table 1**), suggesting a need for intervention.

**Table 1:** T's eating disorder diagnosis, PARDI scores, and weight at baseline and end of treatment.

	Baseline	End of Treatment
**EDA-5 diagnosis**	ARFID	OSFED
**PARDI severity score**	2.18	1.71
**PARDI sensory subscale**	1.50	0.50
**PARDI loss of interest subscale**	0.91	1.64
**PARDI fear subscale**	0.30	0.00
**Weight in kilograms (kg)**	28.00	31.07
**% EBW**	96.95	97.62

EDA-5, Eating Disorder Assessment for DSM-5 [EDA-5; (6)] PARDI, Pica, ARFID, Rumination Disorder Interview (PARDI) (7), a new measure designed to assess ARFID symptom severity; EBW, expected body weight, calculated using Center for Disease Control metrics in children and adolescents (8).

### Phase 1

T presented for FBT-ARFID with her mother, father, and younger brother (age 7). In the first session, the therapist used circular questioning, a strategy where families members are asked to comment on the statements made by previous family members (5) to engage the family in crafting a narrative of the impact of ARFID on their family. T was unable to eat with her family given anxiety and disgust she felt around the foods they served. T's mother felt “tired of being a short order cook,” always preparing a second separate meal for T after she prepared a meal for the rest of the family. The family spent “enormous time and effort” obtaining and preparing food for T to take with her to school and to friends' homes so that she could participate in these activities without going hungry. The family expressed frustration regarding T's being “stubborn” and “unwilling” to consider new foods no matter the growing impact on herself and the family.

The therapist used this information to help the parents to externalize ARFID as an illness rather than blame T for her eating problems. To illustrate the seriousness of ARFID and emphasize the need to help T overcome the disorder, the therapist highlighted the multiple impacts of ARFID on the child and family including impacts on growth, pubertal development, and relationships. The therapist also provided psychoeducation regarding the processes by which ARFID is maintained and thus not likely to remit unless the family takes action. As the family's anxiety heightened, the therapist transitioned into charging the parents with the task of challenging ARFID and framing them as T's best hope for recovery. The therapist asked the family to complete an Always, Sometimes, Never list (3), a form used to classify foods into those the child will eat regularly (“Always”), those their child can eat but with difficulty (“Sometimes”), and those that would be very difficult for the child to eat but very helpful, important, or meaningful if the child were able to do so (“Never”).

In session 2, as instructed by the therapist, the family brought a meal to the session that included items from the Always, Sometimes, and Never categories. The parents chose sandwiches and explained that normally, T's sandwich would be different than everyone else's, but for this meal they prepared her the same sandwich as everyone else. This meant that the sandwich included an array of items across T's Always, Sometimes, and Never categories, and the sandwich itself was a Never item. The family also brought some of T's Always foods as a “backup” (pita bread, cheese). T's father explained that he assumed T would never really eat the sandwich they brought given “we've tried a million times before.” Therapist invited the family to try something different today and decide together what they'd like to see T eat in order to take a meaningful step toward challenging ARFID. Parents decided that they'd like her to have a bite of the sandwich; therapist coached parents to decide on a specific, observable amount they meant and invited them to work together to help her do so.

Initially, the father tried rationalizing with T about why it was important for her to eat, while the mother tried gently asking T to please eat it. Throughout these efforts, T sat with arms folded glaring angrily at her parents, then moved to sitting hunched on the floor under the table in protest. Therapist used T's behaviors to externalize the illness, helping the family see these regressive behaviors as clear signs they were challenging ARFID (rather than signs that T was being stubborn, manipulative, or immature). Therapist attempted to help parents identify strategies they were using that tend to be effective, recognize strategies they were using that were not working well, and try new strategies in their place. Specifically, therapist praised mother's strategy of keeping the food close to T and not letting her avoid it. Therapist also asked father whether reasoning with T tended to work (no) and asked mother whether asking T to eat usually worked (no); therapist then coached parents to clearly state to T the behavior they wanted to see (e.g., “please take a bite” or “you need to eat your sandwich”). After time passed without T budging, father unilaterally informed T that she could earn screen time if she ate what was expected of her. Therapist interrupted and coached parents to discuss and decide on this strategy together before introducing it to T; parents agreed that if T ate a bite, she would earn 15 minutes of iPad time when she got home that evening. T initially did not react, but mother persisted in repeating their behavioral expectation; therapist coached father to support his wife in increasing pressure against ARFID. Father also tried making T laugh and smile. After a few moments of giggling, T took the bite.

The remainder of Phase I sessions focused on helping the family gain momentum and a sense of agency in their ability to increase T's eating flexibility. Each session, the therapist used circular questioning to help the family learn from their previous week's efforts, reinforcing strategies they were using that were working well. The therapist used the Always, Sometimes, Never list to measure progress as well as help the family plan what foods they would like to help T eat next. The therapist also provided consultation regarding the use of behavioral strategies and asked specific questions to help the family make a concrete, pragmatic plan for making progress toward their goals each week. The family prioritized entrees typically prepared in their home, fruits, and vegetables. Toward this end, parents gradually increased the level of challenge of foods they presented T in increasing approximations of their ultimate goals. For example, given T would only eat vegetables that were completely pureed, the family gradually presented to her vegetables that were less and less pureed, via soups and smoothies. T's family chose to continue using contingency plans and found them effective. For example, they offered T a daily food challenge, completion of which earned her 15 minutes of screen time. Initially, T completed her challenges only after lengthy time periods and crying, but after parents instituted a time limit for the food challenge, T soon was able to complete them most days within 15 minutes. Throughout this phase, T's brother's involvement was limited but important; the therapist framed him as a vital support to T as parents took on the task of challenging ARFID. The family worked together to brainstorm and monitor ways in which T's brother was supporting her, from agreeing to color with her after a tough meal, to distracting her with jokes at the dinner table, to not interfering with his parents' efforts even when they evoked distress from T.

### Phase 2

Once T's parents were feeling empowered and T had been able to consume new foods consistently, treatment moved to Phase 2. In this phase, focus shifted to helping T eat a wider variety of foods outside the specific context and strategy the family had found effective (at home, in the afternoon, as part of a daily contingency). For example, family wanted T to be able to eat at extended family gatherings and friends' houses. During this phase, T became a more willing participant in the sessions, giving her parents feedback about ideas they were considering and making requests for foods she would be more interested in trying. As such, one new strategy the family was able to use during this phase was involving T in food preparation and cooking; T found this fun, and being able to prepare her own food challenges and food for the family seemed to help her feel more excited about eating and “proud” of her accomplishments. The family completed treatment after 17 sessions across 6 months. At termination, T was able to regularly eat several vegetables in their original forms (not pureed), several dishes that were staples in the family's home, any soup (did not need to be blended), and three fruits. They added a total of 13 new foods to her Always list. Please see **Table 1** for objective measures of clinical improvement.

## Discussion

This case highlights how FBT-ARFID maintains the key interventions central to FBT for AN and bulimia nervosa (BN). Specifically, as in other forms of FBT, there was no focus on the cause of dysfunctional eating behaviors, in order to reduce any parental guilt or blame and to help the family stay focused on taking action (*agnosticism*). Secondly, the parents were the agents of change, rather than the child or the therapist (*parental empowerment*). Although the therapist often facilitated the family's decision-making and learning and shared insights from expertise, the therapist was never prescriptive (*consultative stance*). The therapist also supported the family in maintaining a *pragmatic focus*, i.e. making specific, realistic plans to address eating behaviors (not cognitions). Additionally, the therapist repeatedly separated ARFID from the child (*externalization*), which helped the parents to recognize and challenge ARFID-driven behaviors. The therapist also repeatedly *emphasized the seriousness of ARFID* in order to help the parents maintain enough sense of urgency that they were spurned to persistently take action against an illness they have lived with and accommodated for quite some time, despite it requiring significant time and mental and emotional resources.

Despite using these same key interventions, this case also illustrates how FBT-ARFID differs from FBT for AN or BN. In some cases of ARFID, it can be harder to build a sense of urgency than it is for AN or BN. Unlike the more acute and salient medical concerns of AN and BN, some children presenting for treatment of ARFID, particularly those with sensory sensitivity, may not yet have experienced clear medical or nutritional consequences of the illness (1). Immediate distress and impairment may be primarily in psychosocial realms, as was the case for T. Thus, the urgency and intense scene the therapist orchestrates may need to be focused on preventing future detriment ARFID could cause if parents fail to intervene now (e.g., growth potential, pubertal development, peer and family problems). With AN or BN, or cases of ARFID with more acute onset and impact (e.g., those with fear of aversive consequences) (1), the urgency and intense scene can include more immediate consequences of the illness.

Similarly, throughout Phase I of FBT-ARFID for T, separating the illness from the child was more challenging than when treating AN or BN. ARFID tends to have an earlier onset, and thus many families have been living with it most of the child's life. It is thus often hard for families to envision their child's life separately from the eating problem, and they often view the eating behaviors as an inherent part of their child. This stands in stark contrast to the acute and later onset of AN and BN, which enables parents of children with those illnesses to clearly remember life before the eating disorder, more easily recognize the eating behaviors as part of an illness that has come into their child's life, and more readily see the possibility of returning to life without these eating behaviors if they can address them.

In addition to challenges with separation of the illness and urgency to action, FBT-ARFID also often differs from FBT-AN in that children with ARFID are more able to participate in the treatment and can do so at earlier stages. Many youth with AN wish to maintain their eating disorder (9). During Phase I of FBT for AN it is not advisable for the adolescent to be involved in decisions about eating. Parents often need to ignore children's requests as most of them are counterproductive. However, children with ARFID are less invested in maintaining their illness. Although many are reluctant to participate in treatment and find it difficult, once they accept that their parents are addressing their eating, they are often motivated to “have a say” in setting goals and challenges. For example, in the case presented, T suggested foods she wanted to be able to eat or options she was interested in her parents considering for food challenges.

Lastly, another difference this case demonstrates between FBT-ARFID and FBT for other eating disorders is that certain strategies may be effective in addressing ARFID that would likely not be with AN or BN. Specifically, T's parents were able to very effectively use behavioral contingencies to help T learn to eat a greater variety of foods. However, incentives are seldom enough to overcome fear or strong motivation to maintain the disorder in AN.

It is worth noting that ARFID is quite heterogeneous, and applying FBT-ARFID to patients with fear of aversive consequences and/or low appetite may require adaptations. Although core FBT interventions reviewed here (e.g., parental empowerment) appear relevant across any ARFID presentation, changes to more specific interventions may be warranted, such as the focus of treatment (e.g., weight gain, expanding eating flexibility) and specific instructions given for the family meal; please see case reports from Lock and colleagues (4) for further guidance on treatment considerations across different ARFID presentations. However, the appropriateness of this treatment for many presentations of ARFID (e.g., older adolescents or young adults, patients relying on enteral feeding or all-liquid diets, patients with significant psychiatric or medical comorbidities) is unknown. While preliminary case reports suggest the feasibility and potential utility of FBT-ARFID (4), systematic study of its effectiveness and for whom is needed.

## Conclusion

This case demonstrates how FBT-ARFID may be used to treat ARFID in a preadolescent with sensory sensitivity and how the approach compares with FBT used to treat other eating disorders. Given the limited evidence base for treating ARFID (10, 11) and growing number of youth referred for its treatment (12), further study of this promising approach is warranted.

## Ethics Statement

The studies involving human participants were reviewed and approved by Stanford University Administrative Panels on Human Subjects Research. Written informed consent to participate in this study was provided by the participants' legal guardian/next of kin.

## Author Contributions

KR and JL co-wrote the manuscript. KR served as a therapist to the patient. JL provided supervision of the treatment and created the manual used during treatment.

## Funding

This work was supported by the National Eating Disorders Association, Grant/Award Number: SPO 125881.

## Conflict of Interest

The authors declare that the research was conducted in the absence of any commercial or financial relationships that could be construed as a potential conflict of interest.
